# Effect of Pre-Heating on Enhancing the Anti-Digestive and Antioxidant Properties of Curcumin Rice by Self-Emulsifying Technology

**DOI:** 10.3390/foods14213668

**Published:** 2025-10-27

**Authors:** Chien-Yu Ma, Yi-Chan Chiang, Po-Yuan Chiang

**Affiliations:** Department of Food Science and Biotechnology, National Chung Hsing University, 145 Xingda Road, South Dist., Taichung City 40227, Taiwan

**Keywords:** thermal process, resistant starch, curcumin, self-emulsion, antioxidant

## Abstract

Self-emulsifying delivery systems (SEDSs) generate micro/nanoemulsions within the gastrointestinal tract, enhancing the bioavailability of hydrophobic phytochemicals. Turmeric rice is a staple across Asia, yet the mechanism of curcumin binding with rice remains unclear. This study utilized curcumin SEDS and pre-heating to produce curcumin instant rice (CIR). The CIR maintained its appearance and increased the levels of slowly digestible starch to 26.43–28.38% following steamer steaming (SST). In contrast, cooker water-boiling treatment (CWBT) enhanced resistant starch (16.73–38.24%), curcumin loading (3.77–61.39 mg/100 g), total phenolic content (23.09–193.79 mg GAE/100 g DW), and antioxidant activity. Following 50 min of CWBT, the gelatinization process disrupted the ordered structure of starch, facilitating the formation of amylose–lipid complexes. This process resulted in a maximum resistant starch content of 47.81% and a curcumin loading of 66.50 mg/100 g. Differential scanning calorimetry revealed an endothermic peak for type II crystals (105–130 °C), while X-ray diffraction exhibited V-type patterns, confirming the observed changes. Pre-heating enhanced the curcumin encapsulation and RS of CIR, thereby enhancing its bioavailability relative to conventional turmeric rice.

## 1. Introduction

Turmeric rice is widely consumed in Asian dietary culture, with high consumer acceptance, and often serves as a substitute for white rice, thus holding a strong market foundation and considerable commercial potential. However, with the global prevalence of obesity continuously increasing and the incidence of chronic diseases such as diabetes, hyperlipidemia, and cardiovascular disorders rising, many studies have begun to focus on the digestibility of starch in rice. Based on the enzymatic hydrolysis rate, starch can be categorized into rapidly digestible starch (RDS), slowly digestible starch (SDS), and resistant starch (RS) [1]. Among these, RS escapes digestion in the small intestine and is metabolized by anaerobic colonic microbiota through glycolysis to produce short-chain fatty acids (SCFAs) such as butyrate, acetate, and propionate, which support gut health. In addition, RS delays gastric emptying, stimulates incretin secretion, enhances satiety, and reduces appetite [2,3,4]. RS can be classified into five types, among which RS5 is a novel form consisting of amylose–lipid complexes formed through noncovalent interactions, including hydrophobic forces and hydrogen bonding between the hydrophobic cavity of amylose helices and the hydrocarbon chains of lipids. RS5 exhibits superior thermal stability and produces higher levels of SCFAs, making it the most intensively studied form of resistant starch [5,6,7,8]. With changing dietary demands, there has been growing interest in foods that combine nutritional value with convenience. Instant rice, owing to its convenience, rapid preparation, and long shelf life, has become an expanding industry [9,10]. Instant rice is typically produced through soaking, precooking, and drying. However, during the precooking process, the crystalline and lamellar structures of starch are disrupted, reducing natural RS content while increasing starch digestibility, thereby elevating the risk of obesity and chronic diseases. Thus, strategies to enhance the nutritional value of instant rice have become an important area of research [11,12].

Curcumin, the major curcuminoid in turmeric (*Curcuma longa* L.), is a polyphenolic compound. Curcuminoids, including curcumin, demethoxycurcumin, and bisdemethoxycurcumin, are natural polyphenols extracted from turmeric. Curcumin possesses various pharmacological activities, including antioxidant, anti-inflammatory, anti-carcinogenic, and neuroprotective effects against Alzheimer’s disease. Therefore, it has been widely used in the development of nutraceuticals and functional foods, such as capsules, tablets, and food additives [13,14,15]. Nevertheless, due to its tautomeric structure, relatively high molecular weight, and aromatic rings, curcumin exhibits poor water solubility and is only partially absorbed through the gastrointestinal epithelium, resulting in extremely low bioavailability [16,17]. Numerous strategies have been explored to enhance its absorption, including the use of curcumin nanoparticles, phospholipid–curcumin complexes, and curcumin analogs. Among these, nanotechnology-based approaches, such as self-emulsifying systems, are currently the most widely used to improve curcumin bioavailability [18,19,20,21].

Self-emulsifying delivery systems (SEDSs) represent an effective technique to enhance the bioavailability of lipophilic compounds such as curcumin by improving their solubility, dispersibility, and absorption. SEDSs include self-microemulsifying delivery systems (SMEDSs) and self-nanoemulsifying delivery systems (SNEDSs), which are formulations composed of oils, surfactants, and optional cosolvents. These systems spontaneously form emulsions upon contact with aqueous media, without the need for mechanical agitation [22,23]. Recently, SEDSs have been successfully applied in the development of functional foods to increase the absorption of poorly soluble bioactives [24,25]. Previous research has confirmed the feasibility of curcumin emulsion-based carriers, pointing to the potential for further applications of curcumin emulsions in food systems [19].

Processing conditions, particularly thermal treatments, play a pivotal role in determining the starch structure and bioactive content of instant rice. To the best of our knowledge, this study is the first to combine curcumin self-emulsifying technology with instant rice production. By applying different precooking treatments, curcumin instant rice (CIR) was developed with the aim of enhancing RS content while simultaneously providing antioxidant benefits, thereby establishing a novel functional instant rice product. Most previous research has examined the formation of RS5 via starch granules. This research enhances the resistant starch content in rice grains through the application of nano-sized oil droplets containing curcumin, which are generated by a self-emulsifying system.

## 2. Materials and Methods

### 2.1. Materials and Chemicals

Tainan No. 11 rice was purchased from Zhongdu Cooperative (Taichung, Taiwan). Curcumin extract powder (≥95% purity) was purchased from Hsin Kang Le Co., Ltd. (Taichung, Taiwan). Sunflower oil was obtained from Standard Foods Corporation (Taichung, Taiwan). Polyethylene glycol sorbitan monooleate (Tween 80) and sorbitan monooleate (Span 80) were purchased from Yue Ba Enterprise Co. (Taipei, Taiwan). The Megazyme GOPOD assay kit was purchased from Megazyme International (Bray, Ireland). All other chemicals and reagents were purchased from Sigma-Aldrich (St. Louis, MO, USA). Deionized water with a resistivity of not less than 18.2 MΩ·cm was used for the preparation of all solutions. Commercial products were purchased from Coupang Enterprise Co. (New York, NY, USA).

### 2.2. Preparation of the SEDS

The formulation of the SEDS was based on the method described by Wu and Chiang [26], using varying ratios of oil and aqueous phases. Sunflower oil, Tween 80, and Span 80 were mixed according to the proportions listed in Table 1. The mixtures were vortexed for 30 s and subsequently centrifuged at 5000 rpm for 5 min. Formulations that exhibited a uniform single-phase appearance were selected for the further evaluation of self-emulsifying performance.

### 2.3. Evaluation of SEDS Formulations

#### 2.3.1. Calculation of HLB and SOR


(1) $${\mathrm{HLB}}_{\mathrm{mix}}=\sum_{i=1}^{n}\left(\frac{W_{i}}{W_{\mathrm{total}}}\times {\mathrm{HLB}}_{i}\right)$$

*HLB*_mix_ = The final HLB value of the mixed surfactants.
*HLB*_i_ = The individual HLB values of surfactants.
*W*_i_ = The respective amounts of surfactants (expressed as weight or molar ratio).

(2) $$\mathrm{SOR}=\frac{m_{\mathrm{surfactant\_mix}}}{m_{\mathrm{oil}}}$$

*m_surfactant_mix_* = The total mass of surfactants.
*m_oil_* = The total mass of oil.

#### 2.3.2. Emulsification Assessment

The evaluation of the selected homogeneous SEDS formulations was conducted following the method of Wu and Chiang [26]; a 0.1 mL aliquot of the formulation was added dropwise into 1.3 mL of deionized water under stirring at 100 rpm for 10 min. The emulsification behavior was subsequently assessed according to the criteria described by Shahba et al. [27]. Spontaneity: Emulsions formed within 1 min were classified as Good, those formed within 1–10 min as Moderate, and those requiring > 10 min as Poor. Homogeneity: Emulsions exhibiting a uniform appearance were rated as Good, those showing partial turbidity as Moderate, and those with phase separation as Poor. Dispersibility: Formulations that dispersed rapidly and completely in water were considered Good, those with partially undispersed fractions as Moderate, and those failing to disperse as Poor.

#### 2.3.3. Curcumin Analysis of SEDS Formulation

In total, 2 g of self-emulsifying formulation was weighed and mixed with 0.1 g of curcumin extract, followed by vortex mixing and centrifugation (5000 rpm, 5 min). An aliquot of 0.1 mL of the supernatant was diluted with 0.9 mL of 95% ethanol and subsequently subjected to ultrasonic extraction for 90 min at 25 ± 5 °C (40 kHz, 700 W). Before analysis, the extract supernatants were filtered through a 0.22 μm PTFE syringe filter (Waters Co., Milford, MA, USA) to remove particulates. The curcumin content was determined using high-performance liquid chromatography with an ultraviolet detector (HPLC-UV), which consisted of an autosampler (Model PN5300, Postnova Co., Salt Lake City, UT, USA), a chromatographic pump (Model Chromaster 5110, Hitachi Co., Tokyo, Japan), a column (Mightysil RP-18GP, 250 mm × 4.6 mm i.d., 5.0 μm; Kanto Co., Tokyo, Japan), and a UV–VIS detector (Model Chromaster 5420, Hitachi Co., Tokyo, Japan). The analytical conditions were adapted from the method of Shirsath et al. [28], with slight modifications. The mobile phase consisted of 2% (*v*/*v*) aqueous acetic acid (42.9%), acetonitrile (52.4%), and methanol (4.7%). The analysis was performed at a flow rate of 1.00 mL/min, and an injection volume of 10 μL. The detection wavelength was set at 428 nm. Curcumin (purity ≥ 99.5%) was used as the standard, and the results were quantified to dry weight (mg/g D.W.) using a calibration curve constructed from standard solutions at 1.8, 0.9, 0.45, 0.225, and 0.1125 μg/mL.

#### 2.3.4. Turbidity

The turbidity of the self-emulsion formulation was determined following the method of Wu and Chiang [26], and 0.1 mL aliquot of the formulation was added dropwise into 1.3 mL of deionized water and stirred at 150 rpm for 10 min. The absorbance of the resulting dispersion was then measured at 600 nm using a microplate reader (Model SPECTROstar Nano, BMG Labtech, Ortenberg, Germany).

### 2.4. Preparation of CIR

The curcumin SEDS was mixed with water at a ratio of 1:13 (*w*/*w*) to form an emulsion system, which was then combined with white rice at a ratio of 1.4:1 (*w*/*w*). The mixture was hand-mixed for 1 min and then soaked at 30 °C for 30 min (moisture: 27.64% ± 0.43%) and subsequently subjected to thermal processing using either a steamer (Ruimeng Metal Co., Ltd., Tainan, Taiwan) or an electric cooker (Tatung Co., Ltd., Taipei, Taiwan) for varying durations and sampling at 10, 20, 30, 40, 50, and 60 min. After heating, the samples were dried using a cold air dehydrator (Model 3926TB, Excalibur Dehydrator Co., Sacramento, CA, USA) at 25 °C for 20 h. The dried samples were ground and passed through an 80-mesh sieve, then stored at −80 °C until further analysis. Samples prepared by steamer steaming (SST) and cooker water-boiling treatment (CWBT) were designated SST-CIR and CWBT-CIR, respectively. White rice and cooked rice without the addition of the curcumin self-emulsifying formulation served as controls, designated RR and CR, respectively.

### 2.5. Physical Characteristics of CIR

#### 2.5.1. Appearance

The surface and cross-sectional morphology of CIR were observed using a stereoscopic dissecting microscope (Model SMZ800, Nikon Corporation, Tokyo, Japan) and documented with a digital single-lens reflex camera (Model 450D, Canon Inc., Tokyo, Japan).

#### 2.5.2. Scanning Electron Microscopy (FE-SEM)

CIR samples were mounted on specimen stubs using conductive double-sided adhesive tape and coated with a thin layer of gold using an ion sputter coater (Model JEC-3000FC, JEOL Ltd., Tokyo, Japan) under a current of 10 mA. The cross-sectional morphology of the CIR samples was examined using a field emission scanning electron microscope (FE-SEM; Model JSM-7800F Prime, JEOL Ltd., Tokyo, Japan) operated in low-vacuum mode at an accelerating voltage of 3 kV.

#### 2.5.3. Color Analysis

The lightness value (L*), green–red value (a*), and blue–yellow value (b*) of CIR were measured using a color meter (Model ZE-2000, Nippon Denshoku Industries Co., Ltd., Tokyo, Japan) and calibrated with a standard white plate (X = 92.81, Y = 94.83, Z = 111.71) and a black box (X = 0, Y = 0, Z = 0). The color difference (Δ*E*) and whiteness index (W.I.) were calculated according to Equations (3) and (4).

(3) $$\Delta E=\sqrt{({L_{1}^{*}-L_{0}^{*})}^{2}+({a_{1}^{*}-a_{0}^{*})}^{2}+({b_{1}^{*}-b_{0}^{*})}^{2}}$$

(4) $$W.I.=100-\sqrt{{\left(100-L^{*}\right)}^{2}+{a^{*}}^{2}+{b^{*}}^{2}}$$

where *L*_1_*, *L*_0_*, *a*_1_*, *a*_0_*, *b*_1_*, and *b*_0_* denote the L*, a*, and b* of the control and samples, respectively.

#### 2.5.4. Water Activity

The water activity of CIR was measured using a water activity meter (Model Aqualab 3TE, Meter Group Inc., Pullman, WA, USA).

#### 2.5.5. Swelling Power and Solubility

Following and modifying the method by Zhu et al. [29], 1 g of CIR was immersed in 10 mL of deionized water and heated at 98 ± 2 °C for 30 min. After heating, the supernatant was separated, and the remaining residue was weighed to calculate the water absorption ratio using the following equation (Equation (5)). The supernatant was transferred to a crucible and dried at 105 °C until constant weight. The dry residue was weighed, and solubility was calculated according to Equation (6).

(5) $$\mathrm{Swelling}\mathrm{power}\left(\%\right)=[\frac{\mathrm{Wc}-\mathrm{Wuc}}{\mathrm{Wuc}}]\times 100$$

(6) Solubility (%)=Ws/Wuc×100

where Wuc, Wc, and Ws denote the weight of the sample before heating, after heating, and the supernatant, respectively.

### 2.6. Starch Structural of CIR

#### 2.6.1. Rapid Visco Analyzer (RVA)

In total, 2.5 g of CIR powder was mixed with 22.5 g of deionized water in an RVA aluminum canister. The pasting properties were measured using a Rapid Visco Analyzer (Model RVA-Ezi, Newport Scientific Pty Ltd., Warriewood, NSW, Australia). The test was initiated at 50 °C with a stirring speed of 160 rpm. After equilibrating for 1.5 min, the sample was heated to 95 °C at a rate of 12 °C/min and held at 95 °C for 2 min. It was then cooled to 50 °C at the same rate and held for an additional 6 min.

#### 2.6.2. Differential Scanning Calorimetry (DSC)

The thermal properties of CIR were analyzed using a differential scanning calorimeter (DSC 1 STAR System, Mettler-Toledo, Greifensee, Switzerland). Briefly, 3.2 mg CIR was weighed and mixed with 12.8 mg of deionized water in a 40 µL aluminum pan (ME-2277331, Mettler-Toledo, Greifensee, Switzerland), then sealed and equilibrated at 4 °C for 24 h. The sample was subsequently heated from 30 °C to 130 °C at a rate of 10 °C/min. The onset temperature (T_o_), peak temperature (T_p_), conclusion temperature (T_e_), gelatinization temperature range (Δ*T*), and enthalpy change (Δ*H*) were recorded.

#### 2.6.3. In Vitro Digestibility

Following and modifying the methods of Englyst & Cummings, Goñi et al., and Canxin Cai et al. [1,30,31], 50 mg of CIR powder was dispersed in 2.5 mL of deionized water, followed by the addition of 1.5 mL of sodium acetate buffer (0.5 M, pH 5.2) and 2.5 mL of guar gum solution (prepared by dissolving 0.01 g of guar gum and 0.001 g of pepsin in 20 mL of 0.05 M HCl). The mixture was incubated at 37 °C for 20 min to simulate gastric conditions. Subsequently, 2.5 mL of an enzyme mixture was added, consisting of 32 mL of supernatant obtained by dissolving 1.267 g of α-amylase in 40 mL of deionized water and centrifuging, combined with amyloglucosidase (121 μL) diluted in 4.879 mL of deionized water. The enzymatic digestion proceeded for 180 min at 37 °C. At the end of the reaction, 168 μL of the digestion mixture was withdrawn and mixed with 0.5 mL of 95% ethanol to terminate enzymatic activity, followed by centrifugation at 3000 rpm for 10 min. Then, 20 μL of the supernatant was reacted with 600 μL of GOPOD reagent at 50 °C for 20 min, and the absorbance was measured at 515 nm. Glucose standards (purity ≥ 99.5%) were prepared at concentrations of 1.8, 0.9, 0.45, 0.225, and 0.1125 μg/mL to construct a calibration curve quantified to dry weight (mg/g). The starch hydrolysis data (sampling at 0, 5, 10, 15, 20, 30, 45, 60, 90, 120, and 180 min) were fitted to a first-order kinetic equation as follows (Equation (7)):

(7) $$C=C\infty (1-e^{-kt})$$

where C is the percentage of starch hydrolyzed at time t, C∞ is the percentage of starch hydrolyzed after 180 min, k is the kinetic constant (min^−1^), and t is the time (min). Starch hydrolyzed within 20 min was defined as rapidly digestible starch (RDS), starch hydrolyzed between 20 and 180 min as slowly digestible starch (SDS), and starch remaining undigested after 180 min as resistant starch (RS).

#### 2.6.4. Complexing Index

Following and modifying the method of Paramee Chumsri et al. [32], 0.4 g of CIR powder was mixed with deionized water to a total weight of 5 g and heated for 10 min. After heating, 25 mL of deionized water was added, and the mixture was vortexed for 2 min. The dispersion was then centrifuged at 4000 rpm for 5 min. An aliquot of 250 μL of the supernatant was mixed with 2 mL of iodine solution (2.0% KI and 1.3% I_2_ dissolved in deionized water) and diluted with 15 mL of deionized water. The absorbance was measured at 690 nm. The complexing index (CI) was calculated using the following equation (Equation (8)):

(8) $$CI(\%)=[\frac{A_{\mathrm{Reference}}-A_{\mathrm{Sample}}}{A_{\mathrm{Reference}}}]\times 100$$

where $A_{\mathrm{Reference}}$ and $A_{\mathrm{Sample}}$ denote the absorbance of the paste containing starch only and of the sample, respectively.

### 2.7. Short-Range and Long-Range Ordered Molecular Sructure Analysis

#### 2.7.1. Fourier Transform Infrared (FTIR) Spectroscopy

FTIR spectroscopy equipped with an MCT detector (Model Nicolet 6700, Thermo Fisher Scientific, Waltham, MA, USA) was used to scan all samples over the range of 650–4000 cm^−1^. The spectra were collected at a resolution of 2 cm^−1^ with an acquisition time of 30 s.

#### 2.7.2. X-Ray Diffraction (XRD)

The crystalline structure of the samples was analyzed using an X-ray diffractometer (X’Pert Pro MPD, PANalytical, Almelo, The Netherlands). The measurements were performed at an operating voltage of 40 kV and a current of 40 mA. Scans were conducted over a 2θ range of 3° to 50° at a scanning rate of 2°/min.

### 2.8. Antioxidant Components and Activities of CIR

#### 2.8.1. Curcumin Content of SEDS Formulation

The curcumin content of CIR extracts, prepared using the extraction method described in Section 2.3.2, was determined using the same HPLC-UV equipment and analysis method as described in Section 2.3.2.

#### 2.8.2. Total Phenolic Content (TPC)

Following and modifying the method of Chiang and Chiang [33], a 70 μL aliquot of the CIR extract was mixed with 70 μL of Folin–Ciocalteu reagent and incubated in the dark for 3 min. Then, 35 μL of 10% (*w*/*v*) sodium carbonate (Na_2_CO_3_) solution was added, and the mixture was vortexed and incubated in the dark for 30 min. The absorbance was measured at 735 nm using a microplate reader. Gallic acid was used as the standard and quantified to dry weight (mg/g) using calibration curves.

#### 2.8.3. DPPH Radical Scavenging Activity

Following and modifying the method by Chuang et al. [34], a 10 μL aliquot of the CIR extract was mixed with 40 μL of Tris-HCl buffer (100 mM, pH 7.4), followed by the addition of 75 μL of 0.5 mM DPPH solution prepared in methanol. The mixture was incubated in the dark for 30 min. The absorbance was measured at 735 nm using a microplate reader. Trolox was used as the standard and quantified to dry weight (mg/g) using calibration curves. The calculation of the DPPH scavenging rate of the samples was performed using the formula below (Equation (9)):

(9) $$\mathrm{DPPH}(\%)=[\frac{A_{\mathrm{blank}}-A_{\mathrm{sample}}}{A_{\mathrm{blank}}}]\times 100$$

where $A_{\mathrm{blank}}$ and $A_{\mathrm{sample}}$ denote the absorbance of the ethanol solution with DPPH solution and the test sample with DPPH solution, respectively.

#### 2.8.4. ABTS^+^ Radical Scavenging Activity

Following and modifying the method of Subashni Bhoopathy et al. [35], ABTS^+^ stock solution was prepared by mixing 10 mL of 7 mM ABTS solution with 5 mL of 2.45 mM potassium persulfate solution, followed by vortexing. The mixture was allowed to stand in the dark at room temperature for 12–16 h to generate ABTS^+^ radicals. Before use, the ABTS^+^ solution was diluted with Tris-HCl buffer (100 mM, pH 7.4) to an absorbance of 0.700 ± 0.02 at 734 nm. Then, 10 μL of CIR extract was mixed with 240 μL of the diluted ABTS^+^ solution and incubated in the dark at room temperature for 30 min. The absorbance was measured at 734 nm using a microplate reader. Trolox was used as the standard antioxidant, and the results were quantified to dry weight (mg/g) using calibration curves. The calculation of the ABTS scavenging rate of the samples was performed using the formula below (Equation (10)):

(10) $$\mathrm{ABTS}(\%)=[\frac{A_{\mathrm{blank}}-A_{\mathrm{sample}}}{A_{\mathrm{blank}}}]\times 100$$

where $A_{\mathrm{blank}}$ and $A_{\mathrm{sample}}$ denote the absorbance of the ethanol solution with ABTS^+^ solution and the test sample with ABTS^+^ solution, respectively.

### 2.9. Statistical Analysis

All results are expressed as mean ± standard deviation (*n* = 3). Statistical analysis was performed using a one-way analysis of variance (ANOVA) with SPSS software (version 12.0, IBM Corp., Armonk, NY, USA). Duncan’s multiple range test (DMRT) was used to determine significant differences among the means. A *p*-value of less than 0.05 was considered statistically significant. Principal component analysis (PCA) and agglomerative hierarchical clustering (AHC) were performed using XLSTAT software (XLSTAT 2023.3.0, Addinsoft Co., New York, NY, USA).

## 3. Results and Discussion

### 3.1. Miscibility of SEDS Formulations

In this study, formulations of different self-emulsifying systems were prepared using different oil-to-surfactant ratios and were subjected to preliminary screening (Table 1). Visual assessment served as the primary criterion for evaluating the quality of the self-emulsifying system, enabling optimization to achieve ideal spontaneity, homogeneity, and dispersibility [36]. The results of the visual evaluation are summarized in Table 2. Formulations with higher hydrophilic–lipophilic balance (HLB) values, ranging from 6.44 to 12.86, exhibited superior self-emulsifying properties, demonstrating at least moderate performance of spontaneity, homogeneity, and dispersibility by effectively reducing the interfacial tension between oil and water. Such reduction facilitates the rapid diffusion of the aqueous phase into the SEDS and promotes the formation of an interfacial film, thereby enabling the spontaneous dispersion of oil droplets into the aqueous phase within a short time to generate moderate/good dispersion and stable oil-in-water (*o*/*w*) emulsions [37].

Among the tested formulations, the ratio of oil:Tween 80:Span 80 at 5:3:2 displayed the most favorable self-emulsifying ability. This SEDS formulation spontaneously formed a transparent emulsion within 1 min, indicating efficient dispersion and stability. Moreover, it exhibited the highest curcumin loading capacity, reaching 7.92 ± 0.04 mg/g (*p* < 0.05), which indicates its potential for effective bioactive delivery. Based on these findings, the formulation with a ratio of oil:Tween 80:Span 80 (5:3:2) was selected for subsequent preparation for CIR.

### 3.2. Physicochemical Properties of Curcumin Instant Rice

**Figure 1:**
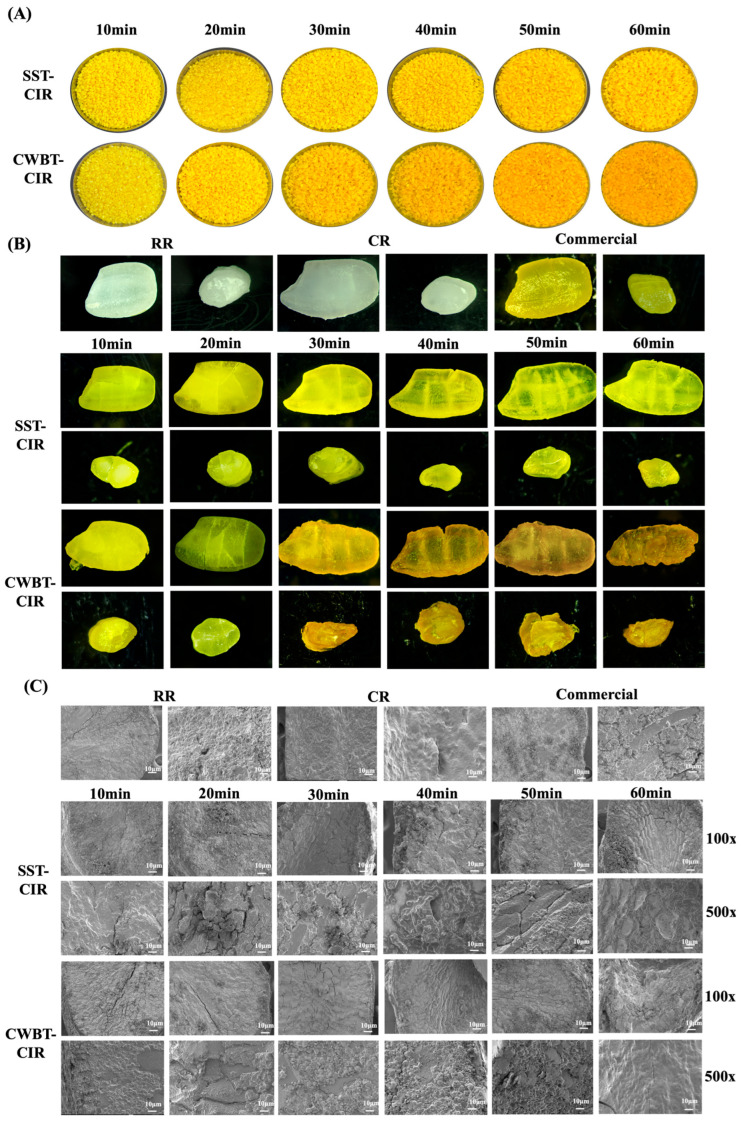
The effects of steamer steaming treatment and cooker water-boiling treatment on the appearance and microstructure of curcumin instant rice. (**A**) Appearance. (**B**) Stereomicroscopic images captured at identical magnifications. (**C**) Scanning electron microscopy (SEM) images at 100× and 500× magnifications. Abbreviations: SST, CWBT, CIR, RR, CR, and Commercial denote steamer steaming treatment and cooker water-boiling treatment, curcumin instant rice, raw rice, cooked rice, and commercial turmeric rice, respectively.

The appearance of rice products is a critical determinant of consumer acceptance, with color uniformity and grain integrity serving as key quality indicators. Thermal processing significantly influences food properties, and previous studies have demonstrated its positive effects on color and texture, such as improving structural characteristics, facilitating the release of natural pigments, and promoting greater color uniformity [9]. Therefore, stereomicroscopy and SEM were employed to examine the surface and microstructural changes of CIR subjected to SST and CWBT. As shown in Figure 1, the appearance of SST-CIR remained largely intact, with uniform color distribution. This observation may be attributed to partial starch gelatinization during SST, which increased porosity and facilitated the diffusion of curcumin emulsion into the grain, thereby enhancing color homogeneity [38,39]. In contrast, CWBT resulted in more pronounced deformation of rice grains, as high temperatures and excessive water diffusion disrupted the granular structure. Starch granules on the surface absorbed water, swelled, and developed cracks, leading to rough surface morphology [40]. Stereomicroscopic cross-sections revealed that the chalky core of CIR gradually diminished with prolonged heating in both SST and CWBT, indicating progressive gelatinization [41]. SEM cross-sectional images further confirmed that CWBT-CIR underwent a greater structural change than SST-CIR. In the early stage of CWBT, native starch granules appeared as smooth, irregular polyhedral structures. However, extended heating caused loosening of the internal matrix, blurring of starch granule edges, and widening of cracks. Moreover, SEM micrographs revealed the presence of larger and irregularly shaped starch granules in both SST-CIR and CWBT-CIR, which are likely attributable to the encapsulation of native starch by the curcumin self-emulsifying formulation [42,43].

**Figure 2:**
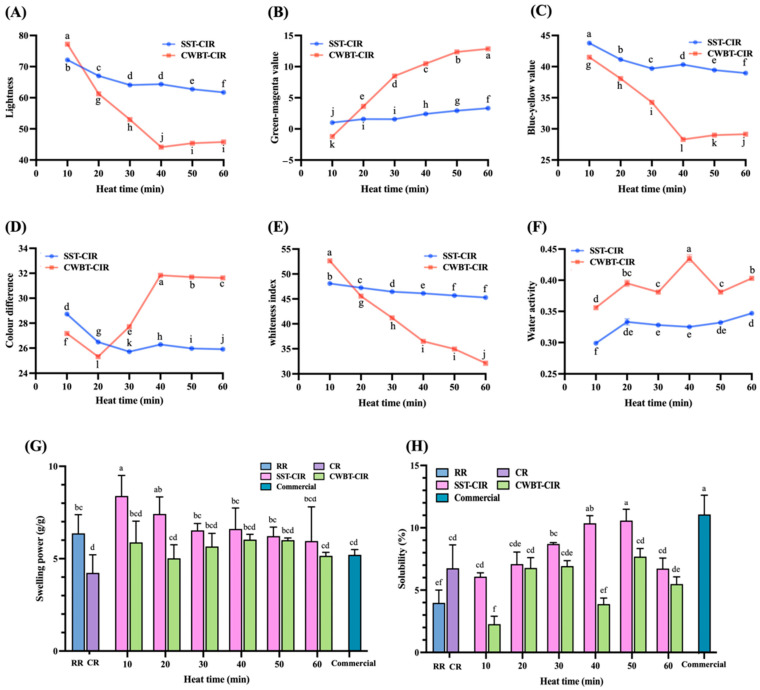
The physicochemical properties of curcumin instant rice include lightness (**A**), green–red value (**B**), blue–yellow value (**C**), color difference (**D**), whiteness index (**E**), water activity (**F**), swelling power (**G**), and solubility (**H**). All values are expressed as mean ± standard deviation (*n* = 3). Different superscript letters (a−l) indicate significant differences (*p* < 0.05). Abbreviations: SST and CWBT denote steamer steaming treatment and cooker water-boiling treatment. CIR, RR, CR, and Commercial denote curcumin instant rice, raw rice, cooked rice, and commercial turmeric rice.

Colorimetric analysis (Figure 2A–C) revealed that L*, b*, and WI decreased after both SST and CWBT, while a* increased, with CWBT-CIR exhibiting more pronounced changes than SST-CIR (*p* < 0.05). The L* and b* of CWBT-CIR decreased from 77.23 ± 0.01 and 41.51 ± 0.02 to 45.76 ± 0.02 and 29.14 ± 0.01, respectively, indicating reductions in brightness and yellowness. Concurrently, a* increased from −1.22 ± 0.02 to 12.86 ± 0.02, reflecting enhanced redness, while WI declined from 52.64 ± 0.01 to 37.12 ± 0.01. These changes were attributed to the diffusion and immersion of curcumin emulsion into rice grains, which altered both its surface and internal coloration [40]. The decreases in L* and b* were also associated with the oxidation or degradation of polyphenolic compounds under thermal treatments [44,45]. The color difference (Δ*E*), an indicator of perceptible color changes, reached maximum values at 40 min of heating for SST-CIR and CWBT-CIR, at 26.69 ± 0.01 and 31.84 ± 0.01, respectively, indicating optimal dyeing effects due to curcumin diffusion at this time point (*p* < 0.05).

Water activity, which influences the reaction rate of lipid oxidation and microbial growth, is a critical parameter for assessing physicochemical and storage stability. Both SST-CIR and CWBT-CIR exhibited water activity values between 0.3 and 0.4 (Figure 2F), a range known to inhibit lipid oxidation and microbial proliferation, thereby indicating well-controlled stability of CIR [46,47]. Figure 2G,H present the swelling power and solubility indices of RR, CR, CIR, and commercial turmeric rice. Starch type, lipid composition, and binding mechanisms influence the swelling power of amylose–lipid complexes, which can indirectly reflect the extent of complex formation. Compared with RR, SST-CIR exhibited higher swelling power [32]. Prolonged heating resulted in a slight decrease in swelling power, likely attributable to the formation of compact starch–lipid complexes via hydrophobic interactions, which hindered water diffusion and the expansion of starch granules. Lipid films on starch surfaces limited water absorption, consequently decreasing swelling power [48]. In contrast, CWBT-CIR consistently exhibited lower swelling power than RR, lacking a clear trend. This was attributed to excessive gelatinization under high-temperature, semi-closed conditions, which disrupted the starch structure and impeded water diffusion [49].

The solubility of both SST-CIR and CWBT-CIR initially increased and subsequently declined with prolonged heating. The initial increase was attributed to starch granule disruption, which released soluble components and facilitated water diffusion into the starch hydrogen bond network [49]. As heating time increased, the slight decline was ascribed to starch molecular rearrangement into denser structures, along with lipid film formation around granules, which restricted water immersion and reduced solubility [43,50].

### 3.3. Rapid Visco Analysis of Curcumin Instant Rice

**Figure 3:**
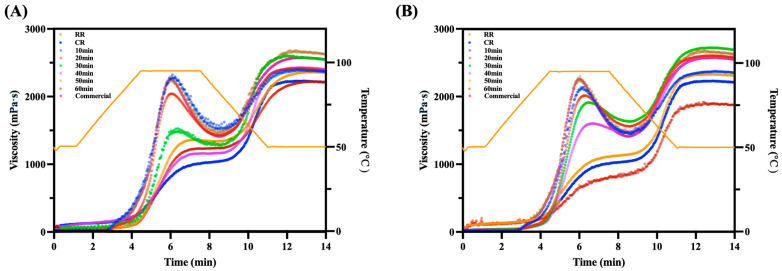
RVA spectra of curcumin instant rice. (**A**) SST-CIR and (**B**) CWBT-CIR. Abbreviations: SST, CWBT, CIR, RR, CR, and Commercial denote steamer steaming treatment and cooker water-boiling treatment, curcumin instant rice, raw rice, cooked rice, and commercial turmeric rice, respectively.

As shown in Figure 3 and Table 3, typical RVA parameters were obtained, including pasting temperature, peak viscosity, trough viscosity, breakdown viscosity, final viscosity, and setback viscosity. During gelatinization, starch granules undergo water absorption, swelling, structural disintegration, molecular leaching, and retrogradation upon cooling. The changes in viscosity during this process reflect the physicochemical transformations of starch.

The RVA results indicated that CR exhibited a peak viscosity of 996.02 ± 10.11 mPa·s and a pasting temperature of 51.70 ± 0.77 °C. In contrast, both SST-CIR and CWBT-CIR displayed higher peak viscosity (2123.94 ± 36.98 and 2142.43 ± 35.37 mPa·s) and elevated pasting temperature (72.30 ± 0.48 and 87.35 ± 0.97 °C) compared with CR [51]. Relative to RR, the peak viscosity of CIR decreased with extended heating; SST-CIR decreased from 2123.94 ± 36.98 to 1234.04 ± 23.04 mPa·s, while CWBT-CIR decreased more sharply from 2142.43 ± 35.37 to 838.38 ± 9.11 mPa·s.

This decrease in viscosity can be explained by starch gelatinization during thermal treatment, which disrupts the granular structure and promotes the leaching of amylose at the initial thermal stage. Prolonged heating allows starch molecules to rearrange and interact with lipids, forming more stable crystalline amylose–lipid complexes. These complexes restrict starch swelling, reduce the leaching of free amylose, and thereby lower overall viscosity [43,50].

During the heating treatment, the pasting temperature of CIR demonstrated an increase in 50 min. Specifically, SST-CIR rose from 87.35 ± 0.97 to 93.85 ± 0.3 °C before slightly decreasing to 92.55 ± 0.4 °C. Similarly, CWBT-CIR increased from 72.30 ± 0.48 to 94.29 ± 0.26 °C, followed by a sharp decline to 54.65 ± 0.55 °C. This pattern suggests that amylose–lipid complexes enhance starch thermal stability by forming compact arrangements, thereby elevating the pasting temperature. However, excessive heating at high temperatures disrupts the crystalline regions of starch, leading to a subsequent reduction in pasting temperature [52,53].

The more drastic fluctuations observed in CWBT-CIR can be attributed to over-gelatinization under high-moisture, semi-closed conditions, which render the starch structure looser and less stable. In contrast, SST-CIR, compared with CWBT-CIR, has more limited water absorption and retained a relatively stable structure, resulting in smaller variations in pasting temperature [54].

### 3.4. Thermal Properties of Curcumin Instant Rice

Table 4 summarizes the onset temperature (T_o_), peak temperature (T_p_), end set temperature (T_e_), and transition enthalpy (ΔH) of RR, CR, and CIR. The crystalline structures of starch–lipid complexes significantly affect their thermal stability, enzyme accessibility, and digestibility, and are generally classified into type I and II crystals. Type I crystals, with melting temperatures between 90 and 105 °C, are formed via rapid nucleation with randomly arranged helical segments. These crystals exhibit a relatively low structural order and are predominantly composed of dispersed single-helical complexes. In contrast, type II crystals exhibit melting temperatures between 105 and 130 °C and are formed under slower nucleation at elevated temperatures. They display a higher structural order with semi-crystalline layered arrangements, characterized by distinct V-type lamellar packing [54,55].

For RR, an endothermic peak appeared at approximately 70 °C, corresponding to starch gelatinization. In contrast, no endothermic peak was detected in CR, indicating complete gelatinization and structural disruption of starch [31]. When starch is heated in the presence of sufficient water, its internal structure transitions from an ordered to a disordered state, accompanied by a significant reduction in enthalpy.

Compared with RR, almost all CIR samples exhibited distinct endothermic peaks within both 90–105 °C and 105–130 °C, corresponding to type I and II starch–lipid complexes, respectively. These results suggest that the incorporation of curcumin emulsion into rice grains facilitated the formation of starch–lipid complexes. Moreover, the transition enthalpy (Δ*H*) of CIR increased significantly with extended thermal treatment, reflecting an increasing quantity of starch–lipid complexes [56]. In particular, the increase in enthalpy associated with type I crystals was relatively smaller compared with that of type II, likely due to the partial transformation of type I crystals into the more ordered type II structure under prolonged heating.

### 3.5. In Vitro Digestibility and Complexation Index of Curcumin Instant Rice

**Figure 4:**
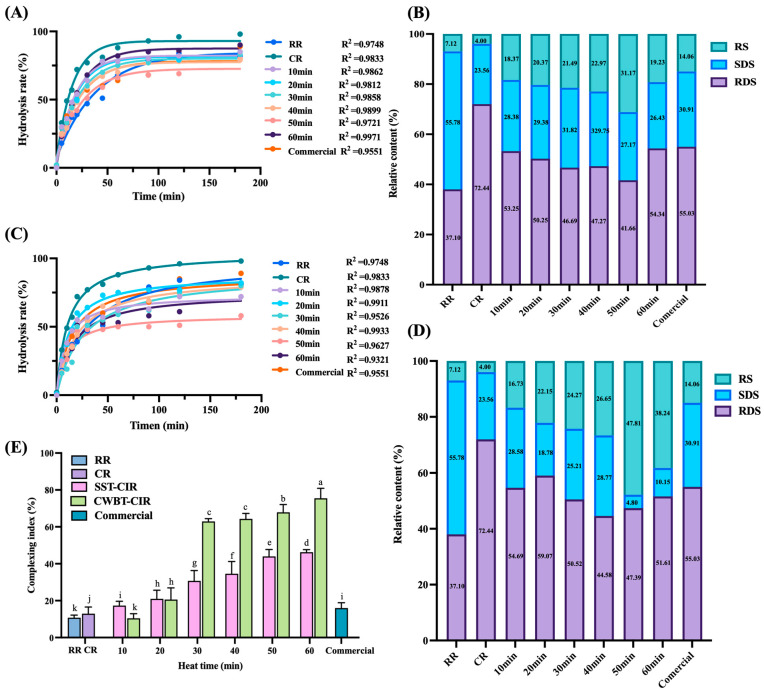
The in vitro digestibility profiles and complexation index (CI) of CIR prepared by different pre-heating processes. (**A**) In vitro digestibility curves of SST-CIR, (**B**) Digestibility rates of SST-CIR, (**C**) In vitro digestibility curves of CWBT-CIR, (**D**) Digestibility rates of CWBT-CIR (**E**) Complexation index. Abbreviations: SST, CWBT, CIR, RR, CR, and Commercial denote steamer steaming treatment and cooker water-boiling treatment, curcumin instant rice, raw rice, cooked rice, and commercial turmeric rice, respectively. Different superscript letters (a–k) within the same row indicate significant differences (*p* < 0.05).

The digestibility of starch can be altered through physical, chemical, or biological modifications, which restrict starch binding to the active sites of α-amylase and amyloglucosidase, thereby reducing enzymatic accessibility and digestibility [51,57]. As shown in Figure 4A–D, RR exhibited the lowest rapidly digestible starch (RDS) content (38.47 ± 2.73%), whereas CR displayed the highest RDS content (72.92 ± 5.47%) and the lowest resistant starch (RS) content (4.09 ± 0.83%). This finding indicates that thermal processing disrupted starch crystallinity, enhancing enzyme–substrate interaction and facilitating starch hydrolysis.

Compared with RR and CR, CIR exhibited a significantly higher RS ratio, which can be attributed to the formation of starch–lipid complexes (RS5). These complexes are stabilized by hydrophobic interactions between amylose helices and exogenous lipids, resulting in compact crystalline structures that inhibit enzymatic hydrolysis [58,59]. CWBT-CIR (1:13 (curcumin SEDS/water), 98 ± 2 °C, 10–60 min) displayed a higher RS ratio than SST-CIR, suggesting that moisture content and temperature play critical roles in RS5 formation [60]. Under high-moisture conditions, gelatinization promotes amylose helix dissociation and leaching, while lipid addition induces conformational changes in the flexible, disordered starch segments. Continuous heating further inhibits excessive amylose leaching and facilitates lipid insertion into amylose helices, yielding stable complexes [60,61].

However, after 50 min of SST or CWBT, the RS ratio decreased (SST-CIR: 31.17% ± 4.38–19.22% ± 3.97%, CWBT-CIR: 47.81% ± 5.37–38.23% ± 5.19%). This decline is due to excessive gelatinization, which disrupted starch double-helical crystallinity and hydrogen bonding within amorphous regions, thereby reducing crystallinity and increasing susceptibility to α-amylase. Thus, although heating initially promoted RS formation, prolonged treatment ultimately reduced RS levels [50,62]. Moreover, CWBT-CIR exhibited a lower slowly digestible starch (SDS) ratio than SST-CIR, likely due to the transformation of loosely ordered type I crystals into more ordered type II structures, thereby decreasing SDS and increasing RS [54]. These findings are consistent with the DSC results (Table 4). Importantly, CIR exhibited higher RS and lower RDS ratios compared with commercial turmeric rice, indicating its potential for moderating postprandial glycemia and supporting functional food development [2,3].

Figure 4E shows the CI values of RR, CR, CIR, and commercial turmeric rice. The CI serves as an indicator of the extent of starch–lipid complexation, as iodine molecules can insert into the helices of free or uncomplexed starch, producing absorbance signals [61]. Both SST-CIR and CWBT-CIR exhibited significantly higher CI values than the controls (*p* < 0.05), suggesting that lipids penetrated starch helices via hydrophobic interactions to form starch–lipid complexes. These results are consistent with DSC and in vitro digestibility findings (Table 4 and Figure 3) [59].

CWBT-CIR exhibited significantly higher CI values than SST-CIR (*p* < 0.05), with the differences increasing over time. This can be explained by the extent of gelatinization. Under SST, heating occurs in a steam-rich but moisture-limited environment, restricting free water availability and limiting starch chain mobility, thereby reducing complexation efficiency [63,64]. In contrast, CWBT provided sufficient moisture, promoting starch porosity and facilitating lipid entry into amylose helices, while continuous heating further enhanced complex formation [40]. Additionally, both SST-CIR and CWBT-CIR exhibited significantly higher CI values than commercial turmeric rice, indicating that these processing methods favor the formation of amylose–lipid complexes in CIR.

### 3.6. FTIR Spectra and X-Ray Diffraction of Curcumin Instant Rice

**Figure 5:**
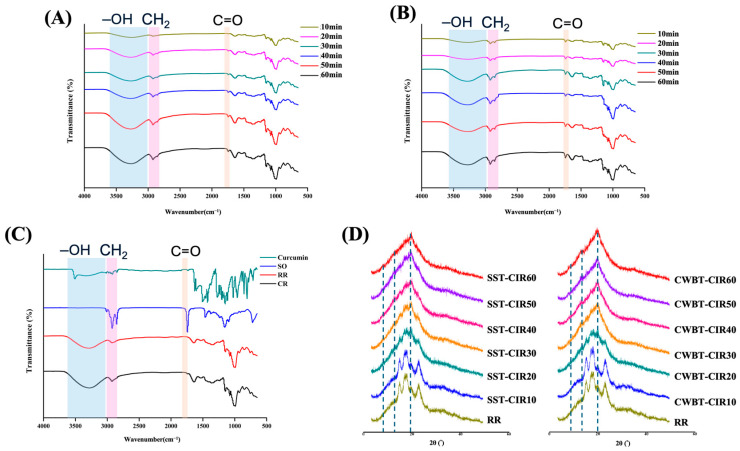
FTIR spectra and X-ray diffraction patterns of curcumin instant rice subjected to different pre-heating processes. (**A**) FTIR spectra of SST-CIR. (**B**) FTIR spectra of CWBT-CIR. (**C**) FTIR spectra of curcumin, sunflower oil, RR, and CR. (**D**) X-ray diffraction patterns of CIR. Abbreviations: SST, CWBT, CIR, RR, CR, and Commercial denote steamer steaming treatment and cooker water-boiling treatment, curcumin instant rice, raw rice, cooked rice, and commercial turmeric rice, respectively. The number after CIR denotes pre-heating treatment time.

ATR−FTIR spectroscopy provides insights into the short-range molecular order of starch. The FTIR spectra of RR, CR, and CIR are presented in Figure 5A–C. The broad band observed at 3000–3600 cm^−1^ corresponds to the O–H stretching vibrations of the amylose and amylopectin hydroxyl groups [65]. With prolonged heating, the O–H stretching region of CIR intensified and broadened, indicating rearrangement of the hydrogen-bonding network. This phenomenon can be attributed to gelatinization-induced disruption of intra- and intermolecular hydrogen bonds in native starch crystals, followed by leaching of amylose during heating. The hydroxyl groups of amylose subsequently formed new hydrogen bonds with surrounding water molecules, leading to structural reorganization and enhanced hydrogen bonding interactions [66].

Compared with RR, CIR exhibited distinct absorption bands at 2850 and 1730 cm^−1^, corresponding to methylene and carbonyl stretching vibrations of fatty acids. These peaks are associated with the formation of RS5 and confirm the establishment of V-type complexes between amylose helices and lipid ligands upon incorporation of curcumin emulsion and subsequent thermal processing [56]. Characteristic absorption bands of curcumin were observed at 3520, 1628, 1510, 1429, 1282, and 1026 cm^−1^, corresponding to –OH, C=O, C–C, aromatic C–O, and C–O–C stretching, respectively. These peaks were less pronounced in CIR, likely due to overlap with starch absorption bands and the limited presence of curcumin as free molecules within the matrix [67].

Previous studies have shown that the absorption bands at 995 and 1047 cm^−1^ are associated with starch crystallinity and ordered structures, while the band at 1022 cm^−1^ represents amorphous regions. Accordingly, the ratios 1047/1022 and 995/1022 are commonly used as indicators for the short-range order of starch [56]. Table 5 summarizes the ratios 1047/1022 and 995/1022 of RR, CR, and CIR. Compared with RR (1.536 ± 0.002), SST-CIR10 exhibited a significantly lower R_1047/1022_ (1.102 ± 0.005), reflecting the disruption of crystalline and double-helical order during gelatinization. However, this ratio increased with extended heating, suggesting that curcumin emulsion accelerates the reorganization of amylose–lipid complexes into more ordered short-range structures [68]. In contrast, the R_995/1022_ of CIR decreased with heating, indicating a reduction in double-helical content, which can be attributed to the binding of lipids with leached amylose chains to form V-type single helices [69].

X-ray diffraction (XRD) analysis is widely applied to characterize starch crystallinity and identify different crystalline types, such as A-, B-, C-, and V-type [54]. Figure 5D shows the XRD patterns of RR, SST-CIR, and CWBT-CIR. RR exhibited typical A-type diffraction peaks at 2θ = 15.2°, 17.3°, 18.2°, and 23.2°. Short-term SST-CIR and CWBT-CIR also displayed similar diffraction peaks, indicating the persistence of native starch crystallinity [69]. However, after prolonged thermal treatment, new diffraction peaks appeared at 2θ = 7°, 13°, and 20°, characteristic of V-type crystals, thereby confirming the formation of RS5 [70]. Prolonged heating disrupted native starch crystallinity, reducing the intensity of A-type peaks due to the disorganization of crystalline and amorphous domains [63]. Simultaneously, hydrophobic interactions promoted lipid insertion into amylose helices, stabilizing V-type complexes. The observed Type A to V polymorphic transition in CIR corroborates the results of the CI measurements [32].

Furthermore, additional diffraction peaks at 2θ = 21.5° and 24° were observed in CIR, generally associated with the presence of free fatty acids [38,71]. With increasing thermal duration, the intensities of the 13° and 20° peaks increased markedly, indicating greater formation of amylose–lipid complexes and enhanced long-range molecular ordering.

### 3.7. Antioxidants and Free Radical Scavenging Activity of Curcumin Instant Rice

Curcumin is a polyphenolic compound that exhibits significant antioxidant activity. This is attributed to its multiple hydroxyl groups, which can donate electrons or hydrogen atoms to neutralize free radicals and reactive oxygen species, effectively interrupting oxidation chain reactions [13,14]. The capacity of antioxidants is significantly associated with various health benefits, such as cardiovascular protection, anti-aging effects, immune system enhancement, and tissue repair. The evaluation of CIR’s functional components and antioxidant activities involved the analysis of curcumin content, total phenolic content, and radical scavenging capacities, specifically DPPH and ABTS^+^.

**Figure 6:**
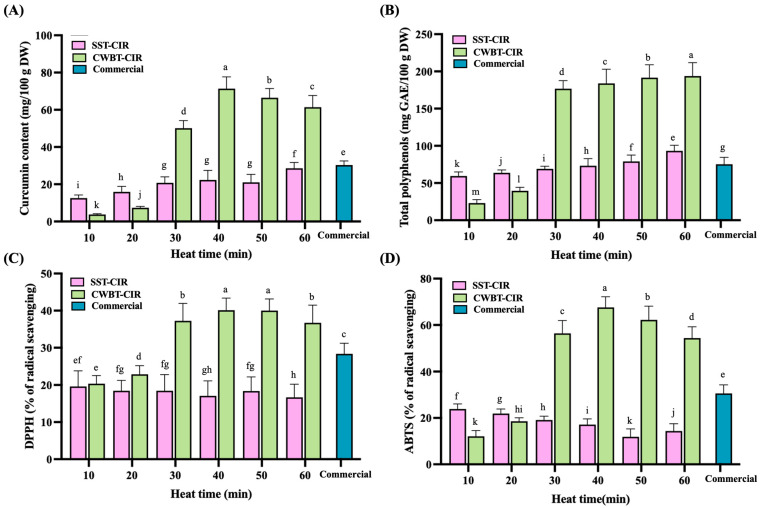
The functional properties of curcumin instant rice. (**A**) Curcumin content. (**B**) Total phenolic content. (**C**) DPPH radical scavenging activity. (**D**) ABTS^+^ radical scavenging activity. All values are expressed as mean ± standard deviation (*n* = 3). Different superscript letters (a–m) within the same row indicate significant differences (*p* < 0.05). Abbreviations: SST, CWBT, CIR, and Commercial denote steamer steaming treatment and cooker water-boiling treatment, curcumin instant rice, raw rice, cooked rice, and commercial turmeric rice, respectively.

As shown in Figure 6A, the curcumin content of CWBT-CIR followed a trend of initial increase followed by a decline, rising from 3.77 ± 0.40 to 71.37 ± 6.29 and subsequently decreasing to 61.39 ± 6.31 mg/100 g DW. This pattern can be attributed to high-moisture thermal processing, which disrupted starch crystallinity and loosened both crystalline and amorphous regions, creating porous structures. The curcumin self-emulsion was dispersed as nanoscale droplets in the aqueous phase, which readily penetrated rice grains, thereby enhancing curcumin loading [18,38]. However, prolonged high-temperature heating caused curcumin degradation into phenolic derivatives, primarily ferulic acid, which further decomposed into vanillin and vanillic acid under moderate heating, leading to reduced curcumin levels [72]. No clear trend was observed in SST-CIR, likely because steam heating under limited water content could not fully disrupt starch structures, restricting curcumin diffusion. Importantly, CIR exhibited markedly higher curcumin content compared with commercial turmeric rice (30.31 ± 2.19 mg/100 g DW), suggesting its potential to provide enhanced health benefits.

Figure 6B illustrates the total phenolic content (TPC) of CIR. Both SST-CIR and CWBT-CIR exhibited significant enhancements in TPC. The observed result is linked to the degradation of curcumin upon heating, leading to the formation of smaller phenolic compounds, including ferulic acid, vanillin, and vanillic acid, which in turn contributed to an increase in TPC [72]. The trend in TPC aligned with the curcumin content, as CWBT-CIR consistently exhibited higher TPC compared to SST-CIR, likely due to its superior curcumin retention.

Figure 6C,D illustrate the radical scavenging activities of CIR. The DPPH and ABTS^+^ scavenging capacities of CWBT-CIR initially increased, were followed by a decline, and were significantly greater than those of SST-CIR. The DPPH scavenging activity increased from 20.34% ± 2.18% to 40.11% ± 3.28%, subsequently declining to 36.72% ± 4.76%. In contrast, the ABTS^+^ scavenging activity rose from 12.08% ± 2.48% to 67.58% ± 4.58%, followed by a decrease to 54.39% ± 4.83%. Both activities peaked at 40 min of thermal treatment, aligning with the trends observed in curcumin content (Figure 6A). Curcumin is a potent antioxidant, and elevated levels during intermediate CWBT durations improve radical scavenging capacity [45]. Prolonged heating, however, results in the degradation of curcumin into phenolic acids, which exhibit comparatively lower radical scavenging efficiency. While degradation products like ferulic acid and vanillin reduce activity, their antioxidant capacities are typically inferior to those of native curcumin, which accounts for the subsequent decline in activity [73].

### 3.8. Principal Component Analysis

**Figure 7:**
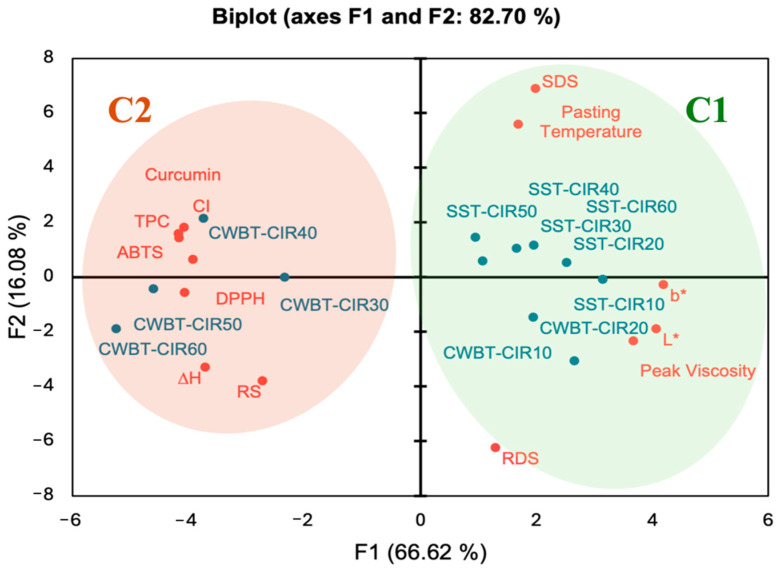
Principal component analysis of curcumin instant rice. Abbreviations: SST, CWBT, CIR, and Commercial denote steamer steaming treatment and cooker water-boiling treatment, curcumin instant rice, raw rice, cooked rice, and commercial turmeric rice, respectively. The number after CIR denotes pre-heating treatment time.

To further investigate the effects of different processing methods and durations on the physicochemical properties and functional components of CIR, PCA was conducted on SST-CIR and CWBT-CIR samples across different heating times. The PCA model incorporated parameters including L*, b*, total enthalpy (Δ*H*), complexation index (CI), RDS, SDS, RS, curcumin content, total phenolic content (TPC), and radical scavenging activities (DPPH and ABTS^+^). As shown in Figure 7, the principal components of F1 and F2 explained 66.62% and 16.08% of the variance, respectively, accounting for a cumulative contribution of 82.70%.

Subsequent AHC analysis divided all CIR samples into two distinct clusters: C1 (SST-CIR10, SST-CIR20, SST-CIR30, SST-CIR40, SST-CIR50, SST-CIR60, CWBT-CIR10, CWBT-CIR20) and C2 (CWBT-CIR30, CWBT-CIR40, CWBT-CIR50, CWBT-CIR60), with significant differences between groups (*p* < 0.05). Cluster 1 (C1) was distributed on the right half-plane of the PCA plot (Figure 7) and was positively associated with peak viscosity, RDS, and SDS. This outcome can be attributed to the limited helix unwinding of starch during steam heating and short heating durations, which were insufficient for forming stable amylose–lipid complexes [48,74].

Cluster 2 (C2) was located on the left half-plane, reflecting the influence of higher moisture content and extended heating time on enhancing the overall functional components and antioxidant capacity of CIR. Moisture is a prerequisite for gelatinization and amylose leaching, which facilitate amylose–lipid complex formation [75,76]. Furthermore, hydrothermal treatment promoted curcumin entrapment within the loosened starch matrix following gelatinization, enabling higher retention of curcumin and total phenolics, thereby enhancing antioxidant activity [38]. Taken together, the PCA and AHC results indicate that the C2 samples exhibited superior functional composition and antioxidant performance, underscoring their potential nutritional and functional value.

## 4. Conclusions

This study investigated the functional properties of curcumin instant rice (CIR) prepared using different pre-heating processes based on a self-emulsifying drug delivery system (SEDS). The results demonstrated that CWBT for 50 min effectively enhanced resistant starch content, bioactive components, and antioxidant capacity. These findings demonstrate that nanodroplets of curcumin emulsion facilitate the formation of thermostable amylose–lipid complexes after CWBT treatment, leading to improved functional properties. Principal component analysis further revealed that CWBT-CIR was associated with lower digestibility and higher antioxidant activity, providing additional evidence that high-moisture processing promotes lipid insertion into amylose helices and enhances curcumin diffusion. In conclusion, CIR combines nutritional and health-promoting benefits, underscoring its potential as a promising functional food with significant development and market value.

## Figures and Tables

**Table 1:** Miscibility screening of sunflower oil with Tween 80 and Span 80 in different ratios.

	Oil:Surfactant	Tween80:Span80				
		4:1	3:2	1:1	2:3	1:4
Sunflower oil	2:1	●	●	●	●	○
	3:2	●	●	○	○	○
	1:1	●	○	○	○	○

● and ○ denote insoluble and soluble, respectively.

**Table 2:** Formulation qualities of the self-emulsifying delivery system.

Sunflower Oil	Oil:Tween 80:Span 80							
	10:1:4	3:1:1	15:4:6	15:2:8	5:3:2	2:1:1	5:2:3	5:1:4
HLB	6.44	9.65	8.58	6.44	10.72	9.65	8.58	6.44
SOR	0.50	0.67	0.67	0.67	1.00	1.00	1.00	1.00
Spontaneity	poor	moderate	poor	poor	good	poor	poor	poor
Homogeneity	poor	poor	moderate	poor	good	poor	poor	poor
Dispersibility	poor	moderate	poor	poor	good	poor	poor	poor
Appearance	milky	semi- transparent	semi-transparent	transparent	transparent	semi-transparent	milky	milky
Curcumin (mg/g)	2.70 ± 0.03 ^h^	5.57 ± 0.09 ^d^	4.78 ± 0.03 ^e^	3.19 ± 0.01 ^g^	7.92 ± 0.04 ^a^	6.90 ± 0.03 ^b^	5.90 ± 0.02 ^c^	3.92 ± 0.01 ^f^

All values are presented as mean ± standard deviation (*n* = 3). Different superscript letters (a–h) within the same row indicate significant differences (*p* < 0.05). Spontaneity: good, emulsification within 1 min; moderate, 1–10 min; poor, >10 min. Homogeneity: good, homogeneous emulsion; moderate, partial turbidity; poor, phase separation. Dispersibility: good, rapid, and complete dispersion; moderate, partial dispersion; poor, no dispersion.

**Table 3:** Pasting properties of curcumin instant rice.

	Pasting Temperature (°C)	Peak Viscosity (mPa·s)	Trough Viscosity (mPa·s)	Breakdown Viscosity (mPa·s)	Final Viscosity (mPa·s)	Setback Viscosity (mPa·s)
RR	76.55 ± 0.61 ^g^	2256.49 ± 35.11 ^a^	1602.10 ± 41.37 ^a^	656.81 ± 8.64 ^b^	2546.84 ± 32.52 ^e^	946.02 ± 11.82 ^i^
CR	51.70 ± 0.77 ^j^	996.02 ± 10.11 ^n^	946.08 ± 14.47 ^k^	49.94 ± 9.47 ^n^	2124.88 ± 18.98 ^m^	1178.80 ± 19.02 ^h^
SST-CIR10	87.35 ± 0.97 ^f^	2123.94 ± 36.98 ^d^	1522.39 ± 28.57 ^d^	633.55 ± 15.63 ^c^	2368.72 ± 45.81 ^h^	868.44 ± 5.11 ^j^
SST-CIR20	90.85 ± 0.42 ^de^	2043.28 ± 27.91 ^e^	1458.85 ± 29.94 ^e^	593.58 ± 11.72 ^e^	2384.10 ± 48.61 ^g^	934.58 ± 17.01 ^i^
SST-CIR30	91.75 ± 0.81 ^cd^	1529.38 ± 20.94 ^i^	1103.72 ± 39.02 ^i^	492.84 ± 10.82 ^h^	2551.34 ± 35.67 ^d^	1447.62 ± 25.17 ^c^
SST-CIR40	92.25 ± 0.48 ^bcd^	1163.49 ± 19.10 ^l^	827.03 ± 19.82 ^l^	343.30 ± 9.77 ^j^	2403.84 ± 21.17 ^f^	1583.65 ± 27.72 ^b^
SST-CIR50	93.85 ± 0.33 ^ab^	1363.59 ± 22.47 ^j^	1037.04 ± 28.36 ^j^	333.10 ± 16.47 ^k^	2351.59 ± 35.64 ^j^	1321.02 ± 13.88 ^e^
SST-CIR60	92.55 ± 0.45 ^abcd^	1234.04 ± 23.04 ^k^	951.95 ± 36.37 ^k^	284.38 ± 13.83 ^l^	2214.05 ± 33.83 ^l^	1264.94 ± 20.14 ^g^
CWBT-CIR10	72.30 ± 0.48 ^h^	2142.43 ± 35.37 ^c^	1540.47 ± 25.39 ^b^	602.45 ± 9.39 ^d^	2353.65 ± 39.72 ^i^	813.57 ± 10.97 ^k^
CWBT-CIR20	89.95 ± 0.43 ^e^	2016.59 ± 13.98 ^f^	1450.48 ± 19.23 ^f^	566.03 ± 10.40 ^f^	2577.38 ± 36.35 ^b^	1127.68 ± 14.21 ^h^
CWBT-CIR30	94.29 ± 0.26 ^a^	1912.44 ± 25.11 ^g^	1400.37 ± 38.28 ^g^	512.04 ± 8.08 ^g^	2693.48 ± 40.06 ^a^	1293.77 ± 19.59 ^f^
CWBT-CIR40	93.95 ± 0.67 ^ab^	1603.45 ± 17.43 ^h^	1239.95 ± 18.28 ^h^	403.92 ± 10.73 ^i^	2569.59 ± 42.06 ^c^	1369.92 ± 22.47 ^d^
CWBT-CIR50	93.00 ± 0.71 ^abc^	1085.28 ± 16.01 ^m^	1039.84 ± 18.04 ^j^	45.44 ± 10.62 ^n^	2214.02 ± 37.94 ^l^	1174.18 ± 21.73 ^h^
CWBT-CIR60	54.65 ± 0.55 ^i^	838.38 ± 9.11 ^o^	603.38 ± 20.73 ^m^	238.72 ± 9.36 ^m^	2323.07 ± 25.67 ^k^	1719.69 ± 24.43 ^a^
Commercial	76.89 ± 0.19 ^g^	2193.84 ± 36.11 ^b^	1538.45 ± 37.39 ^c^	693.84 ± 12.47 ^a^	1880.93 ± 23.17 ^n^	342.48 ± 9.38 ^l^

All values are presented as mean ± standard deviation (*n* = 3). Different superscript letters (a–o) within the same column indicate significant differences (*p* < 0.05). Abbreviations: SST, CWBT, CIR, RR, CR, and Commercial denote steamer steaming treatment and cooker water-boiling treatment, curcumin instant rice, raw rice, cooked rice, and commercial turmeric rice, respectively. The number after CIR denotes the pre-heating treatment time.

**Table 4:** Thermal properties of curcumin instant rice.

	Peak I				Peak II				Peak III			
Sample	T_o_ (°C)	T_p_ (°C)	T_e_ (°C)	ΔH (J/g)	T_o_ (°C)	T_p_ (°C)	T_e_ (°C)	ΔH (J/g)	T_o_ (°C)	T_p_ (°C)	T_e_ (°C)	ΔH (J/g)
RR	64.91 ± 0.13 ^a^	71.14 ± 0.01 ^abc^	79.23 ± 0.45 ^bc^	1.72 ± 0.24 ^a^	–	–	–	–	–	–	–	–
CR	–	–	–	–	–	–	–	–	–	–	–	–
SCIR10	63.32 ± 0.07 ^ab^	69.02 ± 0.01 ^bcdef^	76.84 ± 0.17 ^bcde^	1.36 ± 0.07 ^ab^	–	–	–	–	106.95 ± 0.27 ^a^	116.14 ± 0.01 ^ab^	127.02 ± 0.16 ^abc^	0.24 ± 0.04 ^l^
SCIR20	61.09 ± 0.04 ^abc^	72.65 ± 0.01 ^a^	80.46 ± 0.44 ^ab^	0.19 ± 0.01 ^c^	–	–	–	–	105.03 ± 0.27 ^ab^	116.15 ± 0.62 ^ab^	126.41 ± 4.36 ^bc^	0.26 ± 0.02 ^k^
SCIR30	–	–	–	–	–	–	–	–	101.40 ± 0.01 ^b^	110.74 ± 4.14 ^b^	124.01 ± 2.36 ^c^	0.42 ± 0.05 ^j^
SCIR40	–	–	–	–	–	–	–	–	105.41 ± 5.51 ^ab^	115.89 ± 5.32 ^ab^	131.05 ± 1.47 ^a^	0.60 ± 0.05 ^g^
SCIR50	–	–	–	–	89.50 ± 2.87 ^de^	97.44 ± 0.31 ^f^	103.40 ± 2.54 ^c^	0.17 ± 0.01 ^cd^	107.17 ± 3.16 ^a^	116.57 ± 6.18 ^ab^	129.36 ± 0.04 ^ab^	0.56 ± 0.12 ^h^
SCIR60	–	–	–	–	94.55 ± 4.83 ^abcd^	101.45 ± 2.58 ^abcd^	107.36 ± 1.51 ^c^	0.13 ± 0.03 ^cd^	106.88 ± 0.59 ^a^	120.69 ± 0.01 ^a^	127.88 ± 1.90 ^abc^	0.50 ± 0.03 ^i^
CCIR10	61.98 ± 0.29 ^abc^	67.58 ± 0.01 ^ef^	74.21 ± 0.67 ^cde^	0.95 ± 0.67 ^b^	88.66 ± 1.03 ^e^	97.70 ± 0.08 ^f^	106.03 ± 0.30 ^c^	0.15 ± 0.02 ^cd^	105.22 ± 0.55 ^ab^	115.58 ± 0.08 ^ab^	130.31 ± 1.67 ^ab^	0.70 ± 0.14 ^f^
CCIR20	56.74 ± 4.70 ^cd^	70.24 ± 0.70 ^abcde^	74.32 ± 3.16 ^cde^	0.26 ± 0.13 ^c^	95.17 ± 1.28 ^abc^	101.95 ± 2.01 ^abcd^	108.29 ± 1.82 ^bc^	0.12 ± 0.01 ^d^	105.62 ± 0.26 ^ab^	113.29 ± 0.01 ^b^	130.27 ± 0.16 ^ab^	1.11 ± 0.18 ^e^
CCIR30	–	–	–	–	90.01 ± 1.64 ^de^	99.24 ± 0.08 ^def^	116.04 ± 1.02 ^ab^	0.24 ± 002 ^c^	105.55 ± 4.94 ^ab^	112.95 ± 1.58 ^b^	129.60 ± 0.10 ^ab^	1.18 ± 0.85 ^d^
CCIR40	–	–	–	–	95.32 ± 0.71 ^abc^	101.13 ± 0.01 ^abcde^	110.37 ± 0.47 ^bc^	0.23 ± 0.05 ^cd^	106.17 ± 0.88 ^ab^	120.73 ± 3.13 ^a^	127.13 ± 3.14 ^abc^	1.37 ± 0.08 ^c^
CCIR50	–	–	–	–	99.44 ± 0.37 ^a^	103.33 ± 1.61 ^a^	108.74 ± 1.05 ^bc^	0.35 ± 0.07 ^b^	105.95 ± 0.28 ^ab^	116.38 ± 0.01 ^ab^	129.65 ± 1.82 ^ab^	1.64 ± 0.26 ^b^
CCIR60	–	–	–	–	99.73 ± 0.85 ^a^	101.14 ± 0.08 ^abcde^	109.33 ± 0.55 ^bc^	0.51 ± 0.15 ^a^	105.83 ± 0.18 ^ab^	115.60 ± 0.08 ^ab^	130.64 ± 0.67 ^ab^	3.04 ± 0.25 ^a^
Commercial	58.96 ± 1.15 ^bc^	67.80 ± 0.01 ^def^	72.55 ± 4.10 ^de^	1.03 ± 0.12 ^b^	–	–	–	–	104.70 ± 0.59 ^ab^	113.75 ± 0.15 ^b^	129.18 ± 0.27 ^ab^	0.16 ± 0.01 ^m^

T_o_, T_p_, T_e_, and ΔH represent the onset temperature, peak temperature, end set temperature, and transition enthalpy, respectively. “–“ indicates no detectable peak. All values are expressed as mean ± standard deviation (*n* = 3). Different superscript letters (a–m) within the same column indicate significant differences (*p* < 0.05). Abbreviations: CIR, RR, CR, and Commercial denote steamer steaming treatment and cooker water-boiling treatment, curcumin instant rice, raw rice, cooked rice, and commercial turmeric rice, respectively. The number after CIR denotes pre-heating treatment time.

**Table 5:** Short-range ordered structural characteristics determined by FTIR analysis.

	R_1047/1022_	R_995/1022_
RR	1.536 ± 0.002 ^a^	0.956 ± 0.005 ^bc^
CR	1.263 ± 0.003 ^cd^	0.894 ± 0.006 ^e^
SST-CIR10	1.102 ± 0.005 ^d^	0.985 ± 0.004 ^a^
SST-CIR20	1.112 ± 0.004 ^d^	0.952 ± 0.004 ^bc^
SST-CIR30	1.209 ± 0.005 ^cd^	0.935 ± 0.006 ^d^
SST-CIR40	1.223 ± 0.003 ^cd^	0.906 ± 0.006 ^e^
SST-CIR50	1.335 ± 0.001 ^bc^	0.903 ± 0.002 ^e^
SST-CIR60	1.613 ± 0.006 ^a^	0.852 ± 0.002 ^f^
CWBT-CIR10	1.075 ± 0.007 ^d^	0.981 ± 0.006 ^a^
CWBT-CIR20	1.094 ± 0.003 ^d^	0.961 ± 0.007 ^b^
CWBT-CIR30	1.225 ± 0.006 ^cd^	0.943 ± 0.001 ^cd^
CWBT-CIR40	1.456 ± 0.008 ^ab^	0.963 ± 0.006 ^b^
CWBT-CIR50	1.514 ± 0.004 ^a^	0.933 ± 0.004 ^d^
CWBT-CIR60	1.613 ± 0.008 ^a^	0.905 ± 0.003 ^e^

All values are expressed as mean ± standard deviation (*n* = 3). Different superscript letters (a–f) within the same column indicate significant differences (*p* < 0.05). Abbreviations: SST, CWBT, CIR, RR, CR, and Commercial denote steamer steaming treatment and cooker water-boiling treatment, curcumin instant rice, raw rice, cooked rice, and commercial turmeric rice, respectively. The number after CIR denotes pre-heating treatment time.

## Data Availability

The original contributions presented in this study are included in the article. Further inquiries can be directed to the corresponding author.
